# The Summer Is Coming: *nocte* and *timeless* Genes Are Influenced by Temperature Cycles and May Affect *Aedes aegypti* Locomotor Activity

**DOI:** 10.3389/fphys.2020.614722

**Published:** 2020-12-23

**Authors:** Rayane Teles-de-Freitas, Gustavo B. S. Rivas, Alexandre A. Peixoto, Rafaela Vieira Bruno

**Affiliations:** ^1^Laboratório de Biologia Molecular de Insetos, Instituto Oswaldo Cruz, Fundação Oswaldo Cruz, Rio de Janeiro, Brazil; ^2^Department of Biology, Center for Biological Clocks Research, Texas A&M University, College Station, TX, United States; ^3^Instituto Nacional de Ciência e Tecnologia em Entomologia Molecular (INCT-EM)/CNPq, Rio de Janeiro, Brazil

**Keywords:** circadian rhythms, *Aedes aegypti*, seminatural cycles, temperature cycles, circadian gene expression, clock genes

## Abstract

Mosquitoes exhibit activity rhythms, crucial for the transmission of pathogens, under the control of a circadian clock. *Aedes aegypti* is one of the world’s leading vectors. For decades, several studies have linked the rise in ambient temperature with the increase in their activity. Here, we identify candidate genes whose expression is influenced by temperature cycles and may affect *Aedes* locomotor activity. We observed that *timeless* completely lost its rhythmic expression in light/dark, with out-of-phase temperature cycles, and by RNAi mediated knockdown of *nocte*, an important gene for *Drosophila* circadian synchronization by temperature cycles. Thus, *timeless* and *nocte* are important genes for synchronization by temperature cycles in *Aedes aegypti*. To reinforce our findings, we simulated in the laboratory the gradual temperature fluctuations that were as close as possible to daily temperature variations in Brazil. We observed that the activity and the expression of the molecular circadian clock of *Ae. aegypti* differs significantly from that of mosquitoes subjected to constant or rectangular abrupt changes in temperature. We suggest that for understanding the circadian behavior of *Aedes* with possible implications for intervention strategies, the seminatural paradigm needs to replace the traditional laboratory study.

## Introduction

Most organisms are exposed throughout the day to multiple cycling environmental cues. They are able to adapt and anticipate these changes due to the presence of an endogenous circadian clock that regulates their behavioral and physiological rhythms in accordance with external factors, such as light or temperature. Among the insects, the circadian clock of the model *Drosophila melanogaster* is the best studied so far. In fruit flies, the clock is generated by a set of genes that are linked through transcriptional and translational feedback loops. Two of the main components of *Drosophila* pacemaker—*Clock* (*Clk*) and *cycle* (*cyc*)—encode transcription factors that together form the heterodimer CLK-CYC, which activates the expression of *period* (*per*), *timeless* (*tim*), *clockwork orange* (*cwo*), *PAR domain protein 1ε* (*Pdp1*ε) and *vrille* (*vri*) (Hardin, 2011; Rivas et al., 2016).

In *Drosophila*, these molecular cycles are self-sustained but able to use external cues to synchronize with the environment. Light/dark cycles and temperature fluctuations are the major agents of entrainment (Hardin, 2011; Rivas et al., 2016). In *Drosophila*, Cryptochrome (CRY) synchronizes the molecular feedback loop with the light-dark cycle. This flavoprotein binds directly to TIM in a light-dependent manner, which irreversibly commits TIM to degradation via the proteasome (Emery et al., 1998; Stanewsky et al., 1998; Lin et al., 2001; Busza et al., 2004; Dissel et al., 2004). For temperature entrainment however, *no circadian temperature entrainment* (*nocte*) plays the major role in the synchronization of clock gene expression and activity and is expressed in the chordotonal organs (ChOs, peripheral thermo-mechano sensors) (Glaser and Stanewsky, 2005; Sehadova et al., 2009; Chen et al., 2018).

However, what we understand about *Drosophila* cannot be fully extrapolated to other insects. For instance, gene expression or quantitative trait locus (QTL) analyses suggest that *cryptochrome 2* might be important for the differentiation of chronotypes in mosquitoes. However, this gene does not have an ortholog in *Drosophila* (Gentile et al., 2009; Rund et al., 2011, 2013; Leming et al., 2014; Rivas et al., 2018; Hickner et al., 2019). Knowledge about the clock of other insects is relatively poor compared to *Drosophila* and this lack of understanding aggravates public health problems involving the transmission of pathogens by insect vectors such as *Aedes aegypti*, where the clock determines several characteristics of pathogen transmission, such as locomotor activity, blood feeding, insecticide resistance and oviposition (Clements, 1999; Saunders, 2002; Yang et al., 2010).

Much of what is known about mosquito behavior is based on field studies (Clements, 1999; Saunders, 2002). By using RNA interference, we have observed that the knockdown of *Aetim* affected behavior under standard laboratory conditions (Gentile et al., 2013). We also observed a differential hierarchy of light and temperature as *Zeitgebers* in *Culex quinquefaciatus* and *Ae. aegypti*, where the former is more sensitive to light while the latter is more responsive to temperature (Rivas et al., 2018).

The mosquito *Ae. aegypti* is the main vector of Dengue, Chikungunya, and Zika arboviruses. The importance of temperature for the circadian clock of this species has been studied in the laboratory using rectangular temperature conditions (Rivas et al., 2018).More recent studies in *Drosophila* and other insects in natural and seminatural conditions have revealed that circadian behavior is very different from the traditional laboratory profiles (Currie et al., 2009; Yoshii et al., 2009, 2010; Menegazzi et al., 2012, 2013; Vanin et al., 2012; De et al., 2013; Green et al., 2015). We have therefore studied circadian behavior and gene expression of *Ae. aegypti* under seminatural conditions in the laboratory and observe some interesting changes compared to previous more artificial studies. We focus on temperature cycles and the role of *nocte*.

## Materials and Methods

### Mosquitoes

Eggs of *Ae. aegypti* (Rockefeller strain) were kindly donated by IBEx (Instituto de Biologia do Exército, Rio de Janeiro, Brazil). Mosquitoes were reared from egg stage in LD 12:12 under constant 25°C. The females were separated from males while newly emerged and still virgins. In all experiments, we used 1 to 3 days old virgin females.

### Simulation of Light/Dark and Temperature Cycles

To simulate the light/dark cycles with gradual increase and decrease of luminosity during dawn and dusk, we used a computer controlled LED lighting system (HLT Powerbus USB station, Hoenig Lichttechnik Ltd.). All controls followed the manufacturer’s specifications. Light had an increase from 0 to 1,000 lux, from ZT0 to ZT1.5, and remained stable in 1,000 lux from ZT1.5 to ZT10.5. Then it gradually decreased to 0 lux from ZT10.5 to ZT12, and continued in 0 lux from ZT12 to ZT24. Simulations of both seminatural and rectangular temperature cycles were possible due to the system of temperature ramps coupled to the equipment Solab—SL225/334 (Brazil).

### Locomotor Activity Recording

Each mosquito was placed in a 1 × 7 cm glass tube with cotton soaked in a 10% sucrose solution at one end. Additionally, both ends of the tubes were sealed with Parafilm^®^ M (Sigma-Aldrich). As described in previous studies (Gentile et al., 2009, 2013; Rivas et al., 2018), the circadian locomotor activity rhythms of *Ae. aegypti* were recorded automatically using the DAM10 system, a larger version of the Drosophila Activity Monitoring system (Trikinetics, Waltham, MA). The movement of each mosquito was detected by the interruption of an infrared sensor on the monitor. Daily locomotion was recorded during 30 min intervals. As the mosquito activity data was especially variable, we first transformed the data into logarithm values. In fact, because we had many zeros in the data series, we used log (n + 1) and then calculated the mean of the two experiments. We have been using this practice to minimize distortions due to the high activity of some specimens (Padilha et al., 2018). The graphs and double-plotted actograms were made with Excel^TM^ (Microsoft©) and ActogramJ Software (Schmid et al., 2011), respectively.

We measured the free-running period of 10 consecutive days in constant darkness conditions and we used χ2 periodogram algorithm with ActogramJ, as previously described (Liu et al., 1991; Rund et al., 2013; Rivas et al., 2018). For our analysis, we considered only mosquitoes that presented rhythmicity with a power greater than 10. The power has been used as an efficient method to evaluate the consistency of the rhythm. It was defined as the difference between the top of the peak and the confidence level in the χ^2^ periodogram (Liu et al., 1991).

### Expression of Circadian Clock Genes

Female mosquitoes were kept for 3 days in the chosen regimen and on the third day we collected 10 individuals every 4 h for a 24 h period. Each experiment represented six time-point samples, and this procedure was repeated three or four times. The total RNA of the heads was extracted with the TRIzol method (Invitrogen, Carlsbad, CA), and the cDNA was synthesized with TaqMan Reverse Transcription Reagents (Applied Biosystems, Foster City, CA) following the methods described by Gentile et al. (2009). The final cDNA concentration was 1 ng/μL. Then, we made a relative quantification via real-time PCRs (qPCRs), using the Power SYBR Green PCR Master Mix (Thermo Fisher, Waltham, MA) in the StepOnePlus Real-Time PCR System (Thermo Fisher, Waltham, MA). We amplified *per*, *tim*, *cry2*, *cyc*, *Pdp1*, *vri*, *Clk*, and *rp49* genes using oligonucleotides designed by Gentile et al. (2009). For the genes *E75*, *cwo* and *nocte* we designed new ones, as described in Supplementary Table 1. We used *rp49* gene as a constitutive control and measured the relative mRNA abundance with the comparative C_*T*_ method (Pfaffl, 2001). The values obtained for the relative abundance of mRNA were illustrated by Excel graphs.

### RNA Interference (RNAi) Experiments

To promote the knockdown of the *Ae. aegypti nocte*, we followed the RNAi methodology described by Gentile et al. (2013). We amplified and cloned 762 bp of the coding sequence of this gene into a pGEM-T Easy vector (Promega), in accordance with the manufacturer’s protocol (Supplementary Figure 3). Then, we performed a polymerase chain reaction (PCR) with the plasmid containing the cloned *nocte* fragment and oligonucleotides complementary to *nocte* with a sequence of T7 promoter (see Supplementary Table 1), generating a fragment of 540 bp (Supplementary Figure 3). We purified the reaction product with the GFX PCR DNA & Gel Band Purification Kit (GE Healthcare). Then we synthetized the double-stranded RNA (dsRNA) with the MEGAscript kit (Life Technologies). The dsRNA was purified with lithium chloride. Both procedures were in accordance with the manufacturer’s protocols. We quantified the material in the NanoDrop 3300 (Thermo Scientific). To generate the dsRNA of the control *LacZ* gene, the same procedure was conducted, as described by Gentile et al. (2013). The dsRNA, in a concentration of 3.0 μg/μl, was injected into one 3 days old virgin female *Ae. aegypti* using the Nanoject II micro-injector (Drummond Scientific). Each mosquito was injected with 207 nl of material. After injection, the recovered mosquitoes were used in the experiments of locomotor activity or analysis of the *nocte* expression with quantitative real-time PCR. The molecular analyses were performed on the fourth day after the injection with the dsRNAs. For more details, please see Supplementary Figure 3 and Supplementary Table 1.

### Statistical Analysis

First, we tested if all parameters of activity followed a Gaussian distribution with the Shapiro-Wilk normality test (*p* ≥ 0.05). The E peaks of activity in constant darkness (DD) with seminatural or rectangular temperature cycles (TC) were analyzed using the Mann-Whitney *U*-test (*p* < 0.05). The percentage of activity in photophase (ZT0.5–12) and darkphase (ZT12.5–24) of *Aedes aegypti* in different conditions was calculated with the Student’s *t*-test.

We also calculated if the expression relative abundance of each gene varied significantly throughout the 24 h period of each regimen. We considered that a gene would have a rhythmic expression if the mRNA abundance differed significantly among the six time-point samples using a One-Way ANOVA (*p* ≤ 0.05). The expression of *nocte* in mosquitoes injected with dsRNA of *nocte* or dsRNA of *LacZ* was statistically evaluated using the Student’s *t*-test. All statistical analyses were conducted with the GraphPad Prism 5 (Prism, La Jolla, CA).

## Results

### Simulating Natural Temperature and Light/Dark Cycles

**FIGURE 1:**
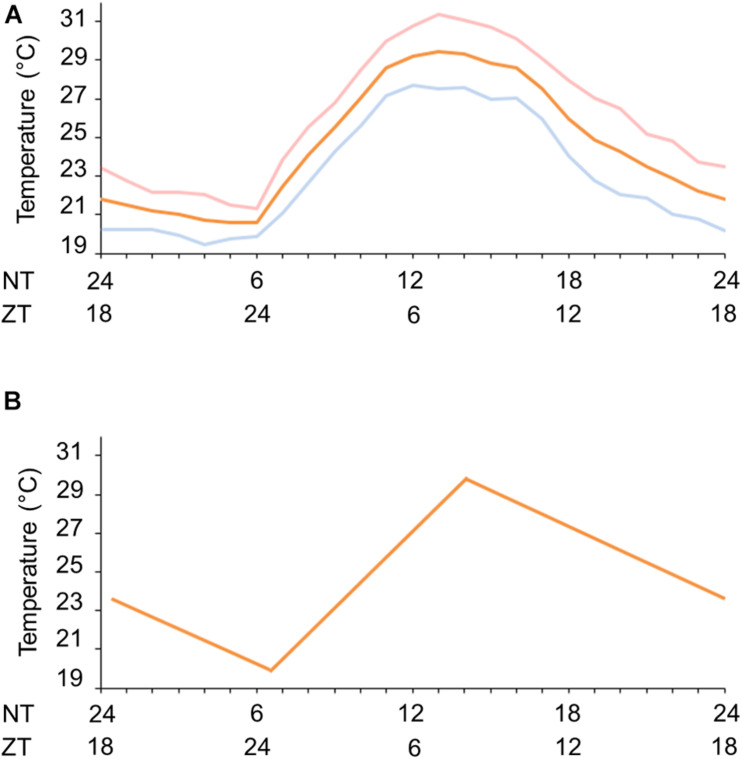
Natural and simulated temperature cycles. **(A)** Natural temperature conditions during fall equinox (pink line), spring equinox (blue continuous line) or the average of equinoxes (orange line) in Rio de Janeiro, RJ, Brazil. **(B)** Gradual simulated temperature cycle (orange line). ZT, zeitgeber time (h); NT, natural time (h) in accordance with the official Brasília time (BRT, UTC-3).

We first set a natural temperature cycle reference with the aid of the National Institute of Meteorology (INMET/Brazil). We obtained the temperature measurements in Rio de Janeiro, RJ, Brazil (A621 weather station, Vila Militar) for average March and September equinoxes from 2008 to 2013. We observed a minimum average temperature of 20.6 ± 1.24°C at 6 a.m. (ZT 0, ZT = *Zeitgeber* time. ZT is the temporal relation of the circadian rhythm to entraining signals such as dawn or the first introduction of light, i.e., wake time) and a maximum average temperature of 29.3 ± 4.92°C at 2 p.m. (ZT 8) (Figure 1A). With this information we were able to simulate seminatural temperature cycles in the laboratory and, based on the gradual temperature fluctuation, we set a minimum of 20°C at ZT 0 and a maximum of 30°C at ZT 8 (Figure 1B).

Defining a natural light/dark cycle reference was more complex because of brightness variations, which involve a range of spectral changes. Secondly, because brightness intensity varies from 0 to 10,000 lux, depending not only on the time of the day, but also on the weather conditions (sunny or cloudy days) and the place (opened or shaded areas),we used as reference a light/dark cycle previously established to analyze *Drosophila* activity under conditions similar to that of an equinox (Rieger et al., 2007; Currie et al., 2009; Yoshii et al., 2009, 2010; Menegazzi et al., 2012, 2013; Vanin et al., 2012; De et al., 2013; Green et al., 2015). Then, we applied an artificial regimen of 12 h of light and 12 h of dark. Light transitions, which mimic dawn and dusk, were conducted using gradual increases or decreases of light. We used a maximum light intensity of 1,000 lux (Gentile et al., 2009; Rund et al., 2011, 2013; Leming et al., 2014; Rivas et al., 2018; Hickner et al., 2019).

### Locomotor Activity Assays in Light/Dark Cycles With Simulated Dawn and Dusk

**FIGURE 2:**
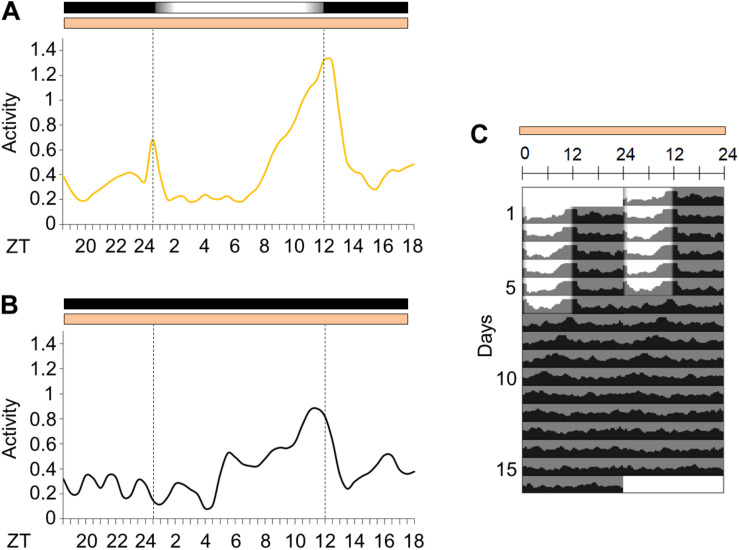
Activity in light/dark cycles with simulated dawn and dusk or in constant darkness. **(A)** Average locomotor activity patterns of *Aedes aegypti* under gradual LD (yellow continuous line). The average pattern was obtained from the average activity from the 3rd to the 5th day in gradual LD. **(B)** Locomotor activity profiles of the first day in constant darkness (black continuous line). **(C)** Average actograms of *Aedes aegypti*. The mosquitoes were kept in gradual LD for 6 days, then light/dark cycles were interrupted (DD) for 10 days (*n* = 14). Temperature remained constant (25°C) every day. Area in actogram indicates light/dark conditions: lights on = white, lights off = gray. Horizontal bars above the average activity profiles lines and actogram indicate light/dark or temperature regimen: lights on = white, lights off = black, 25°C = orange. Dotted lines in average activities profiles indicate the beginning and the end of the photophase in LD or subjective photophase in DD. Error bars are not shown for clarity.

We decided to observe the locomotor activity of *Ae. aegypti* under light/dark cycles with simulated dawn and dusk (from now on considered as “gradual” LD cycle) and constant temperature—LD12:12, 25°C. The mosquitoes were kept in gradual LD cycles for 6 days. They maintained 54% of locomotor activity in the photophase (Supplementary Figure 1A) and showed a bimodal activity pattern with a morning peak (M peak) at dawn, and an evening peak (E peak) at dusk (Figures 2A,C). After 6 days, the LD cycles were interrupted, and the insects were kept under constant darkness and temperature (DD25°C) for additional 10 days. In such conditions, the average locomotor activity apparently became unimodal with a large and broader E peak and a free-running period of approximately 22.2 ± 0.87 h (Figures 2B,C). However, the individual profile showed that 57% of the rhythmic mosquitoes maintained the bimodality even in constant conditions (see Supplementary Figures 2A–D for examples of bimodal and unimodal individuals). The average activity appeared to be unimodal in free-running conditions because the M peak of bimodal individuals was much weaker than the E peak.

### Comparing Seminatural and Rectangular Temperature Cycles

**FIGURE 3:**
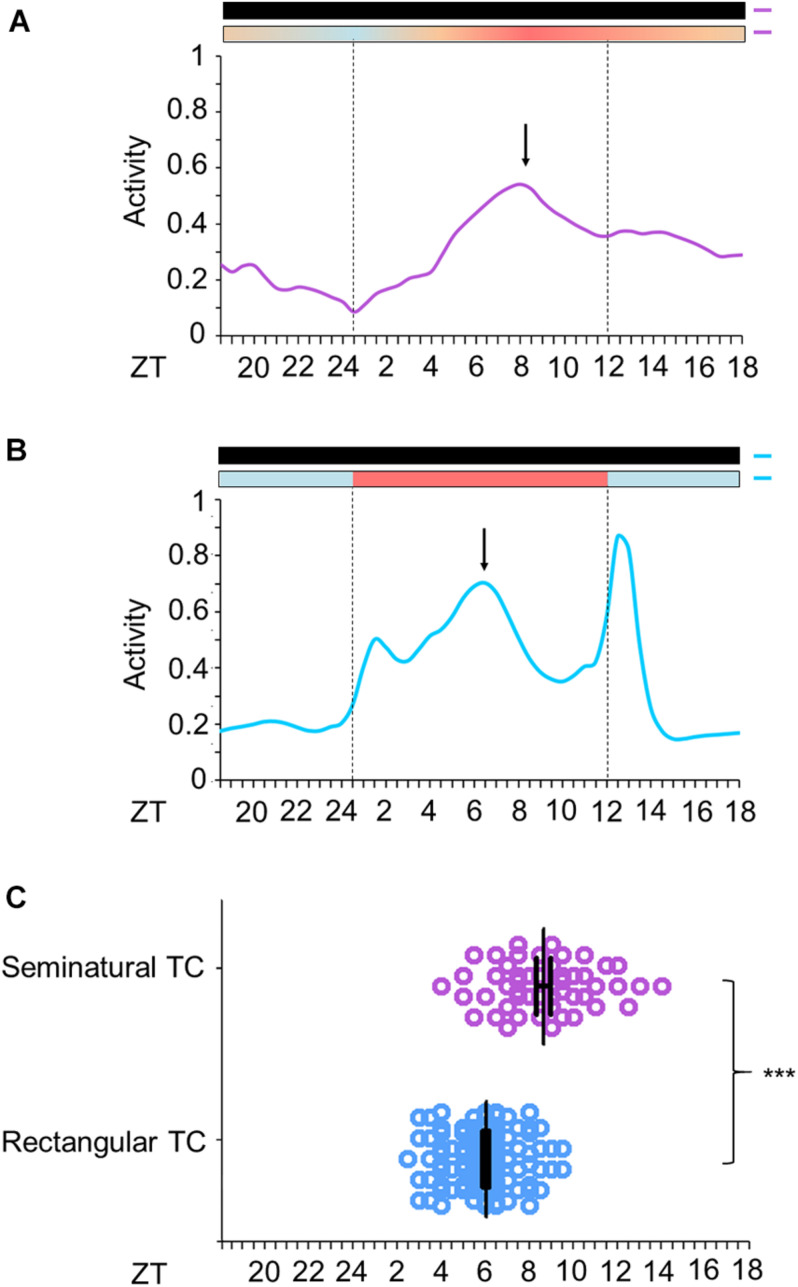
Activity profile of *Ae. aegypti* under seminatural or rectangular TC. **(A,B)** Average activity patterns of *Aedes aegypti* under seminatural TC (**A**, *n* = 51) or rectangular TC (**B**, *n* = 115) and constant darkness. Bars above the average activities profiles lines indicate light/dark or temperature regimen: lights off = black, 20°C = blue, 25°C = orange, 30°C = red. Dotted lines indicate the beginning and the end of the subjective photophase in DD. Arrows present the E peak. Error bars are not shown. **(C)** Analysis of E peak phase of each individual under seminatural TC (ZT 8.65 ± 2.16; lilac circles) or rectangular TC (ZT 6.03 ± 1.54; blue circles) with constant darkness. ***P < 0.001 in accordance with the Mann-Whitney U-test. The average activity patterns or the individual activity profiles used for the analysis of E peaks were both obtained from the average activity from the 4th to the 6th day in each condition.

**FIGURE 4:**
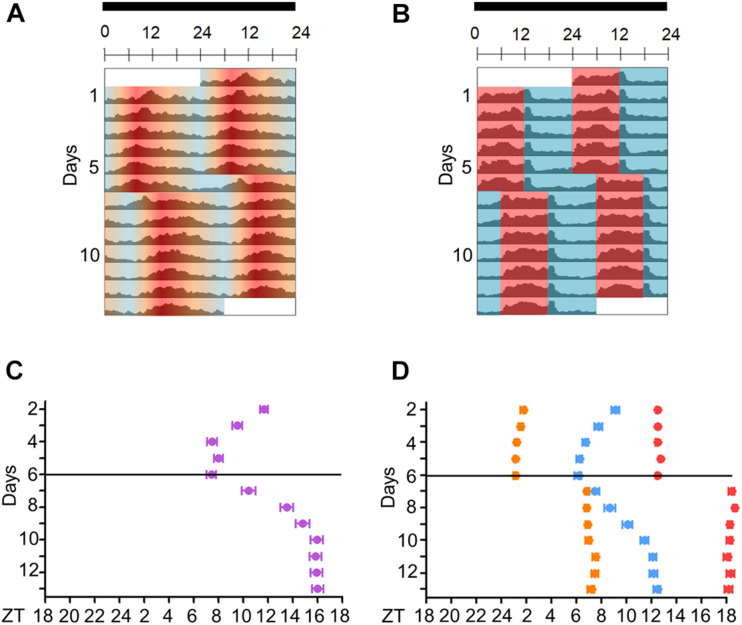
Activity of *Aedes aegypti* over several days in seminatural or rectangular temperature cycles. **(A,B)** Double-plotted actograms of average activity in seminatural TC (**A**, *n* = 51) or rectangular TC (**B**, *n* = 115) with constant darkness. First, the mosquitoes were entrained by each TC for 6 days; then temperature cycles were phase-delayed by 6 h, and they were kept in that new condition for 7 days. **(C,D)** The graphics show the trajectories of the activity peaks of each individual before and after phase-shift of seminatural TC (**C**, *n* = 23) or rectangular TC (**D**, *n* = 30). **(C)** E peak is shown under seminatural TC (lilac dots). **(D)** In rectangular TC, we see the E peak (blue dots) and the peaks that happened just after the temperature rise (orange dots) or temperature drop (red dots). Black bars above the actograms indicate darkness. Areas in actograms represent the temperature cycles: 20°C = blue, 25°C = orange, 30°C = red.

We then compared *Aedes* daily activity in cycles under seminatural temperature conditions to those in rectangular conditions. Mosquitoes were kept in constant darkness (DD) under seminatural or rectangular temperature cycles (TC) for 6 days (Figures 3A–C). Then, each TC was phase-delayed by 6 h and kept in this new condition for 7 days (Figures 3, 4).

Just as the well-known rectangular TC (Figure 3B; Rivas et al., 2018), the seminatural TC was also able to entrain the locomotor rhythms of *Ae. aegypti*. However, there were dissimilarities regarding the locomotor activity profile in each condition. Under the seminatural TC, we observed a single peak of activity (E peak) that remained stable around ZT8 (Figure 3A). On the other hand, in the regimen of rectangular TC, the E peak occurred a little earlier (ZT6). Moreover, a small peak is visible as soon as the warm phase starts and a strong peak following the beginning of the cold phase can be observed (Figure 3B). We believe that the two peaks after the temperature transitions are startle responses induced by the abrupt changes, since they are not observed when the temperature changes gradually (Figures 3A,B).

In addition, when seminatural or rectangular temperature cycles were phase-delayed by 6 h, both TCs were able to re-entrain the locomotor activity. In both conditions, the phase of the main peak of activity indicated that the insects needed about 4 days to be re-entrained to the new phase (Figure 4). In contrast, the other two peaks immediately followed the temperature transitions after the phase-shift of rectangular TC (Figures 4B,D), reinforcing the assumption that they were a clock-independent response to the abrupt transitions.

Thus, a seminatural temperature cycle was able to entrain the activity of *Ae. aegypti*. Our seminatural TC seems to abolish the masking effects of sudden temperature transitions, and may reproduce natural daily rhythms of activity. The entrainment of the behavior are driven by the temporal changes of clock gene expression so we examined the circadian expression of *per*, *tim*, *cryptochrome 2* (*cry2*), *cyc*, *Pdp1*, *vri*, *Clk*, *E75*, and *cwo* mRNAs in the head of *Ae. aegypti* females under seminatural TC with constant darkness.

**FIGURE 5:**
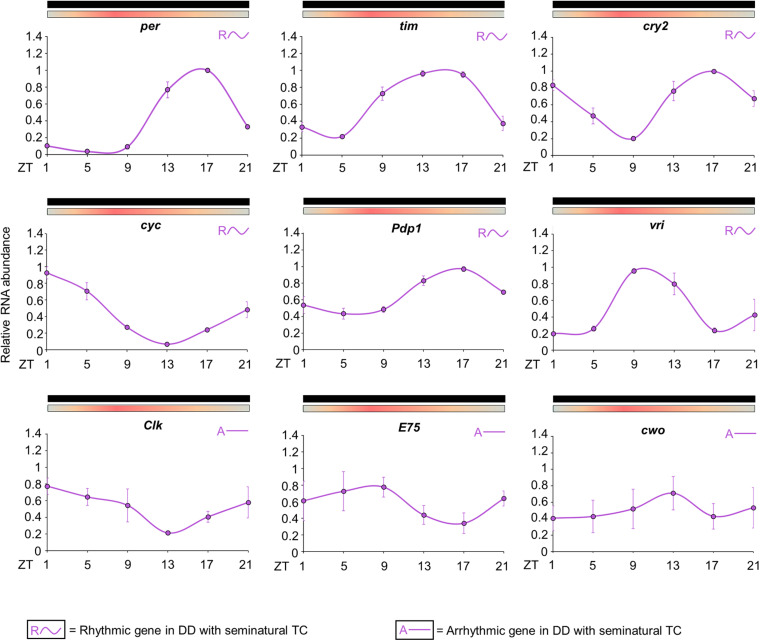
Circadian expression of clock genes in *Ae. aegypti* under seminatural TC with constant darkness. The graphs show the expression of *per*, *tim*, *cry2*, *cyc*, *Pdp1, vri*, *Clk*, *E75*, and *cwo* genes in the head of *Ae. aegypti* under seminatural TC with constant darkness (lilac lines). The average was obtained from data of four independent experiments. The *y*-axis indicates the relative mRNA abundance and the *x*-axis represents the time points (ZT). Bars above the graphs indicate darkness or temperature regimen: lights off = black, 20°C = blue, 25°C = orange, 30°C = red. Rhythmic or arrhythmic genes are identified with “R∼” or “A-” symbols, respectively, according to One-way ANOVA. For more details, see Supplementary Table 2.

The results revealed that the expression patterns are similar to those observed in previous studies for the majority of clock genes (Figure 5; Gentile et al., 2009). The statistical analysis confirmed the rhythmicity of *per*, *tim*, *cry2*, *cyc*, *vri* and *Pdp1* (Supplementary Table 2). *per* showed a peak of expression at ZT17 and a trough at ZT5. *tim* expression is broader than *per* and exhibited a peak at ZT13 and a trough at ZT5. The expression of *cry2* showed a trough at ZT9 and two peaks; the first at ZT1 and the second at ZT17. *cyc* expression exhibited a peak at ZT1 and a trough at ZT13. *Pdp1* and *vri* also presented a cycling profile with a trough at ZT5, but the maximum expression of *vri* occurred earlier. *Pdp1* showed a peak at ZT17, while *vri* peak expression occurred at ZT9. The statistical analysis suggested that *Clk, E75* and *cwo* have no evident rhythmic expression in the whole head (Figure 5 and Supplementary Table 2). Then, as well as observed in the behavioral profile, the circadian expression of clock genes in seminatural TC was not identical to what we knew in rectangular TC. To corroborate this observation, we reanalyzed the expression profile described by Rivas et al. (2018) for rectangular TC. Thus, we could compare the peak and trough expression of the clock genes in seminatural or rectangular TC (Supplementary Tables 3, 4. For more details, see section “Discussion”).

### Synergic Entrainment by Light/Dark and Temperature Cycles

**FIGURE 6:**
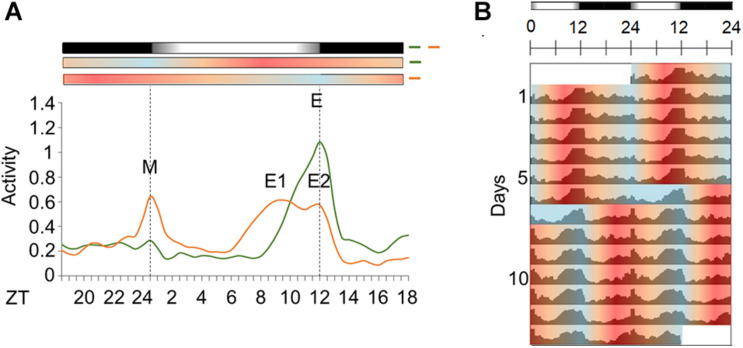
Activity in light/dark cycles with temperature cycles in natural phase or out of phase. **(A)** Average activity profile of *Aedes aegypti* under seminatural LD with in-phase (green continuous line) or out-of-phase (orange continuous line) TC. Dotted lines indicate the beginning and the end of the photophase. Error bars are not shown. M represents the Morning peak and E represents the Evening peak. **(B)** Double-plotted actograms of average activity when mosquitoes were kept in LD with in-phase TC for 6 days, and after phase-shifting by 12 h. The mosquitoes were kept in that new out-of-phase condition for 7 days (*n* = 74). Bars above the actogram or average activities profiles lines represent light/dark cycle: lights on = white, lights off = black. Temperature cycle is indicated by bars above the average activities profiles lines or area in actogram: 20°C = blue, 25°C = orange, 30°C = red. The average patterns were obtained from the activity data from the 3rd to the 5th day before or after phase-shift of seminatural TC.

In nature, light/dark or temperature cycles act synergistically to entrain the circadian clock (Rieger et al., 2007; Gattermann et al., 2008; Currie et al., 2009; Yoshii et al., 2009, 2010; Vanin et al., 2012; Green et al., 2015). Thus, we wondered how seminatural light/dark and temperature cycles would simultaneously entrain the circadian clock of *Aedes aegypti*. To address this question, the mosquitoes were entrained for 6 days in seminatural LD with TC combined in the same phase. On the seventh day, temperature cycles were shifted by 12 h and the mosquitoes were kept in that “out-of-phase” condition for 7 days (Figures 6A,B).

Under LD with in-phase TC, the mosquitoes maintained 56% of their activity during the day (Supplementary Figure 1B), and we could see the onset of an intense activity at ZT8. This activity progressively increases until its maximum at ZT12 (E peak). After lights-off, the activity decreased and remained low throughout the dark phase. Moreover, the mosquitoes exhibited a robust E peak and an almost imperceptible M peak (Figures 6A,B).

A previous report showed that *Ae. aegypti* has a prominent and unexpected nocturnal activity under rectangular light/dark with out-of-phase temperature cycle (Rivas et al., 2018). We investigated if it occurred when seminatural light/dark and temperature cycles are conflicting. The results demonstrated that activity remained diurnal even in seminatural conflicting conditions (66% of activity during the photo phase, Supplementary Figure 1C). On the other hand, the pattern of activity changed remarkably. More specifically, there was an increase in the M peak and a decrease in the E peak, when we compared the graphs for both regimens. Furthermore, the former E peak splits into two peaks. One shifts to a little earlier, at ZT8.5 (E1 peak), while the other remains at ZT12 (E2 peak). This made the onset of the E peak occur earlier than usual, while the offset was not modified (Figures 6A,B).

**FIGURE 7:**
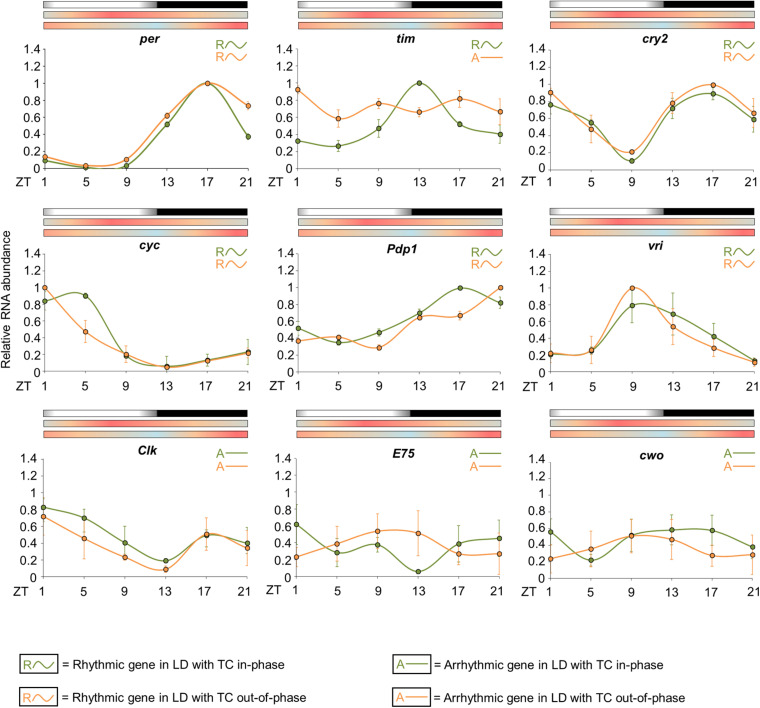
Circadian expression of clock genes when *Zeitgebers* are in phase or conflicting. The graphs display the relative RNA abundance of *per*, *tim*, *cry2*, *cyc*, *Pdp1, vri*, *Clk*, *E75*, and *cwo* in head of *Ae. aegypti* under seminatural LD with in-phase (green lines) or out-of-phase (orange lines) TC. The average was obtained from data of four independent experiments. Bars above the graphs indicate light/dark or temperature regimen: lights on = white, lights off = black, 20°C = blue, 25°C = orange, 30°C = red. Arrhythmic or rhythmic genes are labeled with “A-” or “R∼” symbols, respectively, in accordance with the One-way ANOVA. The color of each symbol indicates the regimen: green = LD with TC in-phase, orange = LD with TC out-of-phase. For more details, see Supplementary Table 2.

Our group recently showed that the circadian expression of clock genes can be modified when rectangular LD and TC are conflicting, compared to rectangular in-phase conditions (Rivas et al., 2018). We speculate whether the same expression changes would be observed under more natural conditions. Thus, we compared the circadian expression of *per*, *tim*, *cry2*, *cyc*, *Clk*, *vri*, *Pdp1*, *E75*, and *cwo* in the head of females when seminatural cycles of light/dark and temperature were combined in-phase or out-of-phase (Figure 7).

The statistical analysis confirmed the rhythmicity of *per*, *cry2*, *cyc*, *vri*, and *Pdp1*, while *Clk*, *E75*, and *cwo* appeared to be arrhythmic in either seminatural conditions (in-phase or out-of-phase) (Supplementary Table 2). We did not observe significant differences regarding the expression profiles of *per*, *cry2*, *vri*, *Clk*, *E75*, and *cwo* under conflicting and reinforcing conditions (Figure 7). In both circumstances, *per* has an expression peak at ZT17 and a trough at ZT5. We noted only an increased expression at ZT21 under seminatural conflicting conditions. *per*, *cry2* and *vri* had their phase unaltered when we compared the regimens. The rhythmic abundance of *cry2* mRNA presented one peak at ZT1, another one at ZT17 and a trough at ZT9. *vri* exhibited a peak at ZT9 and a trough at ZT21 (Figure 7).

In contrast, the circadian expression of *cyc* and *Pdp1* was phase-shifted. *cyc* transcription presented a trough at ZT13 and a peak at ZT5 when the insects were under seminatural LD with in-phase TC. In seminatural conflicting conditions, *cyc* rhythmic abundance remained with a trough at ZT13, but advanced its peak to ZT1. The circadian expression of *Pdp1* was phase-delayed under the out-of-phase regimen compared to in-phase conditions. *Pdp1* mRNA exhibited a peak at ZT17 and a trough at ZT5 under seminatural LD with in-phase TC. When LD and TC were out-of-phase, *Pdp1* showed a peak at ZT21 and a trough at ZT9 (Figure 7).

However, *tim* expression shows the most dramatic changes under the different conditions. When the mosquitoes were under seminatural LD with in-phase TC, *tim* showed a peak at ZT13 and a trough at ZT5. Though, *tim* rhythmic expression was completely abolished under seminatural LD with out-of-phase TC (Figure 7).

### *nocte* and the Entrainment by Temperature Cycles in Mosquitoes

Since seminatural temperature cycles could directly affect the circadian clock of *Ae aegypti*, we wondered which input pathways are responsible for entraining the mosquito clock oscillators by temperature cycles. One possible candidate is *nocte*. As already mentioned, this gene is pivotal for the entrainment by temperature cycles and for the functionality of the chordotonal organs in *D. melanogaster* (Sehadova et al., 2009; Chen et al., 2018). Therefore, we decided to evaluate the role of *nocte* in temperature entrainment using RNA interference.

**FIGURE 8:**
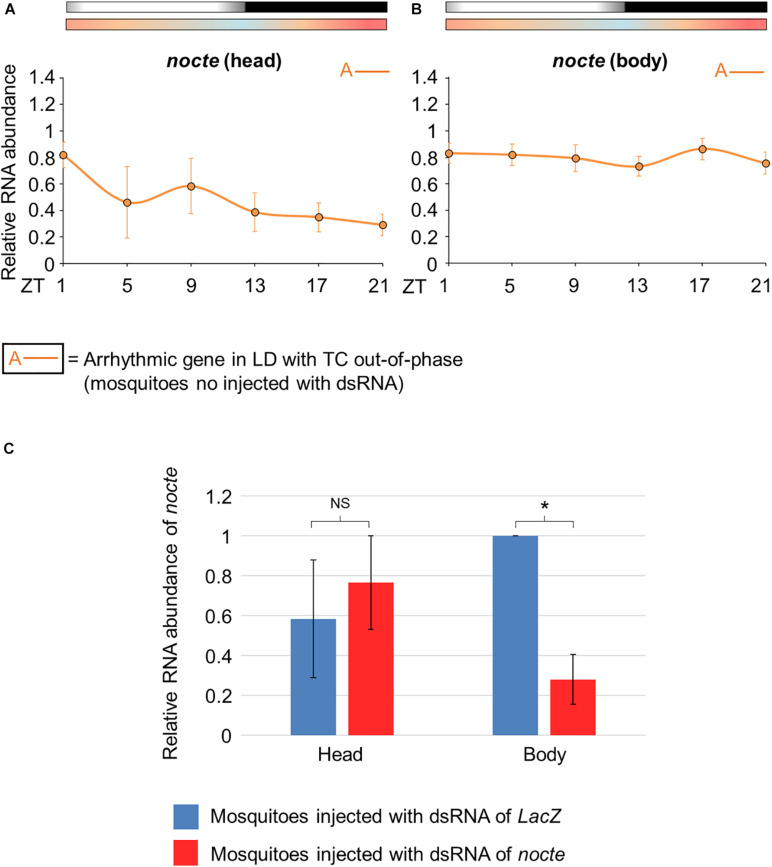
Relative RNA abundance of *nocte* in *Aedes aegypti.* **(A,B)** Pattern of daily transcriptional expression of *nocte* in the head **(A)** or body **(B)** of mosquitoes under seminatural LD with out-of-phase TC (mosquitoes no injected with dsRNA). The average was obtained from data of four experiments. Bars above the graphs indicate light/dark or temperature regimen: lights on = white, lights off = black, 20°C = blue, 25°C = orange, 30°C = red. According to the One-way ANOVA, *nocte* was arrhythmic in the head and body (labeled with “A-” symbol, further details in the text). **(C)** Expression of *nocte* at ZT 21, in the head and body of *Aedes aegypti* injected with dsRNA of *LacZ* (blue) or *nocte* (red) and submitted to LD with out-of-phase TC. The analysis were performed on the fourth day after the injection with the dsRNAs. The experiments were repeated three times and the highest value between the two groups is applied as reference. A *t*-test was used to compare the groups in each tissue. Asterisks indicate when RNA abundance is significantly different among groups (*P* < 0.05). NS, non-significant different of RNA abundance between groups.

Before confirming the silencing of *nocte*, we carefully analyzed its expression profile in uninjected mosquitoes. *nocte* expression was arrhythmic in the head and in the body under the conditions to which the mosquitoes were subjected, i. e., LD with out-of-phase TC (Figures 8A,B). It was also observed in the head of under LD or DD with constant temperatures (Leming et al., 2014). Next, we compared mosquitoes injected with ds*nocte* to the control group (*dsLacZ*) on the fourth day after injection at ZT21. We confirmed the efficacy of the RNAi in reducing *nocte* expression in the body (Student’s *t*-test, *t* = 5.8; *p* < 0.05), but not in the head (Student’s *t*-test, *t* = 0.37; *p* = 0.74) of mosquitoes injected with *nocte* dsRNA (Figure 8C).

**FIGURE 9:**
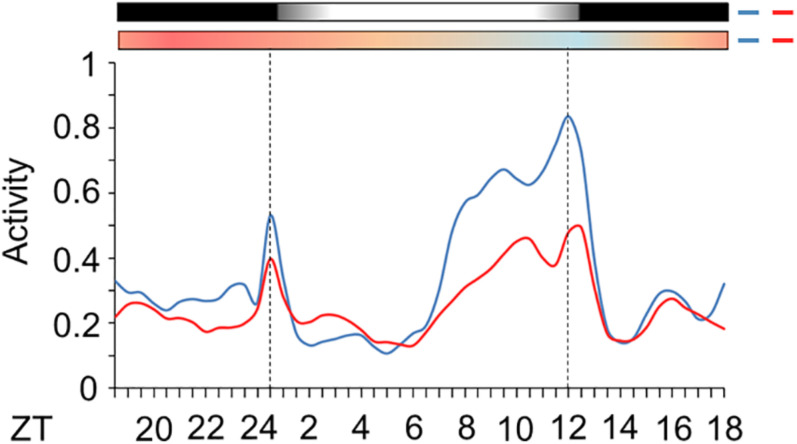
Activity of *Ae. aegypti* injected with dsRNA. The graph shows the activity profile of mosquitoes injected with dsRNA of *LacZ* (blue line) or *nocte* (red line). The average patterns were from the activity data from the 3rd to the 5th day in LD with out-of-phase TC. Bars above the graphs indicate light/dark or temperature regimen: lights on = white, lights off = black, 20°C = blue, 25°C = orange, 30°C = red. Error bars were omitted for clarity. Dotted lines indicate the beginning and the end of photophase.

Subsequently, we evaluated the activity of mosquitoes injected with *nocte* or *LacZ* dsRNAs under seminatural LD with out-of-phase TC. This regimen might reveal how the silencing of *nocte* could affect the amplitude of activity and onset of the E peak, two characteristics highly influenced by seminatural temperature cycles in *Ae. aegypti* (Figure 6A). Both groups remained with a diurnal profile, since there was 59 or 61% of activity in photophase for mosquitoes injected with *LacZ* dsRNA or *nocte* dsRNA, respectively (Supplementary Figures 1D,E). The mosquitoes injected with *nocte* dsRNA exhibited the same offset of the E peak as those injected with *LacZ* dsRNA (Figure 9). The onset of the E peak was not modified either, although there was a greater individual variability in the group injected with *nocte* dsRNA (data not shown). On the other hand, the amplitude of activity was reduced in silenced mosquitoes (Figure 9).

*nocte* and chordotonal organs may be crucial to the entrainment by temperature cycles of *Ae aegypti*, but how would the chordotonal organs communicate with the central pacemaker? We investigated this by comparing the circadian expression of *per*, *tim*, *cry2*, *cyc*, *Clk*, *vri*, *Pdp1*, *E75*, and *cwo* in the head of females injected with dsRNA of *nocte* or *LacZ* under seminatural LD with out-of-phase TC.

**FIGURE 10:**
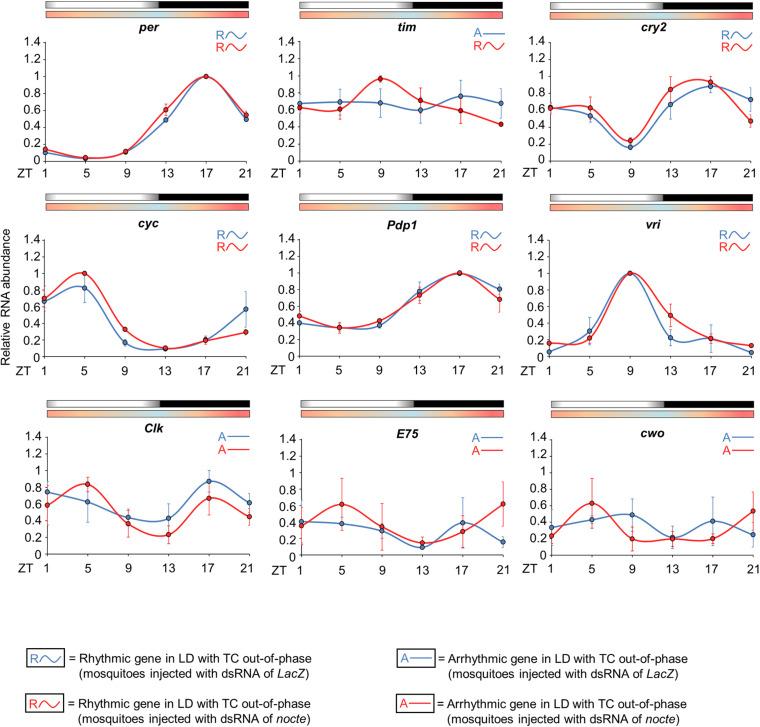
Circadian expression of clock genes in *Ae. aegypti* injected with dsRNA. The graphs show the expression of *per*, *tim*, *cry2*, *cyc*, *Pdp1, vri*, *Clk*, *E75*, and *cwo* in the head of *Ae. aegypti* injected with dsRNA of *LacZ* (blue line) or *nocte* (red line). The mosquitoes were submitted to LD with out-of-phase TC. The average was obtained from data of three independent experiments. All analyzes were performed on the fourth day after the injection with the dsRNAs. Bars above the graphs indicate light/dark or temperature regimen: lights on = white, lights off = black, 20°C = blue, 25°C = orange, 30°C = red. Arrhythmic or rhythmic genes are labeled with “A-” or “R∼” symbols, respectively, in accordance with the One-way ANOVA. For more information, see Supplementary Table 2.

Interestingly, the expression profile of clock genes was very similar in both groups, with the exception of *tim*. The statistical analysis confirmed rhythmicity of *per*, *cry2*, *cyc*, *vri*, and *Pdp1*, whereas *Clk*, *E75* and *cwo* were arrhythmic in mosquitoes injected with *LacZ* dsRNA and in those injected with *nocte* dsRNA (Supplementary Table 2). In both groups, *per* and *Pdp1* presented a peak at ZT17 and a trough at ZT5. The *cry2* gene had a trough at ZT9 and showed two peaks at ZT1 and ZT17. *cyc* exhibited a peak at ZT5 and trough at ZT13, and *vri* presented a peak at ZT9 and a trough at ZT21 (Figure 10). It is worth noting that the mosquitoes injected with *nocte* dsRNA were able to present a rhythmic expression for *tim* even in LD with out-of-phase TC, while the mosquitoes injected with *dsLacZ* lost rhythmicity. The gene *tim* exhibited a peak at ZT9 and a trough at ZT21 when *nocte* was silenced (Figure 10). This was a further indication that *tim* could be crucial for the entrainment of the circadian clock by temperature cycles.

## Discussion

For decades, the behavior of insect vectors, like mosquitoes have been conducted in the field, where a correlation between the capture rates and the activity at a particular time was established (reviewed by Clements, 1999; Saunders, 2002; Taye et al., 2016). Although those studies contributed significantly to our current knowledge, they had some limitations and one of them was the population density throughout the year. In addition, with such methodological approaches, the average activity of a population could conceal individual variations and it would be more difficult to investigate the entrainment by each *Zeitgeber* individually.

In order to mitigate these problems, several techniques were developed to evaluate individual mosquito activity in the laboratory (Taylor and Jones, 1969; Jones, 1981; Gentile et al., 2009; Padilha et al., 2018). However, the majority of studies were conducted in light/dark conditions with constant temperature. In addition, in the reports in which the activity of mosquitoes in temperature cycles was evaluated, such conditions were conducted using cycles with abrupt changes of temperature (Rivas et al., 2018; Upshur et al., 2019). In this study, we simulated the gradual increase or decrease of light at dawn and dusk, and standardized a seminatural regimen to be used in laboratory conditions, which would be as close as possible to the natural temperature cycles of Rio de Janeiro (Figures 1A,B).

We noticed that female *Ae. aegypti*, which were kept in a simulated dawn and dusk LD conditions, sustained their diurnal behavioral rhythms with morning and evening peaks of activity (Figures 2A,C). This pattern is similar to what has been seen for locomotor activity of *Ae. aegypti* in LD with abrupt changes of light (Gentile et al., 2009). Observing the activity of these mosquitoes in simulated dawn and dusk LD is relevant since in *D. melanogaster* the moments of light transition presumably are the most critical hours of the day for the synchronization. For instance, pulses of light coinciding with these moments are enough to entrain activity rhythms of *D. melanogaster* (Pittendrigh, 1964; Sheppard et al., 2014). In a simulation of dawn and dusk with their respective gradual changes of light intensity, the flies adjust their morning and evening peaks of activity when the light is close to 7.5 lux. Furthermore, gradual transitions at dawn and dusk eliminate the undesirable startle responses induced by the abrupt changes in rectangular light/dark cycles (Rieger et al., 2007; Yoshii et al., 2009).

Thus, as in *Drosophila*, we noticed that the gradual LD used could yield the characteristic bimodality pattern in *Aedes*. After this regimen, the period of activity in constant conditions was very much like the period of locomotor and flight activities of female *Ae. aegypti* in DD (Taylor and Jones, 1969; Jones, 1981; Gentile et al., 2009; Figures 2B,C). In addition, we observed that about half of the individuals presented a bimodal activity pattern even in DD. This proportion had also been found in virgin females of *D. melanogaster* (Canton-S strain) (Helfrich-Forster, 2000). Curiously, the studies that investigated the locomotor and flight activities of female *Ae. aegypti* described a unimodal pattern in DD (Taylor and Jones, 1969; Jones, 1981; Gentile et al., 2009). We suppose there are two possible reasons for this. Firstly, the authors of the previous studies probably did not observe a bimodal profile because their analysis was focused on the evaluation of the activity patterns of the mosquito based on actograms or graphs using the mosquito average activity, which is similar to our average activity pattern that is also unimodal (Figures 2B,C). Secondly, as in previous conditions, the days preceding the constant darkness were rectangular LDs (Taylor and Jones, 1969; Jones, 1981; Gentile et al., 2009) and here we simulated dusk and dawn days before transferring the mosquitoes to DD, we do not rule out the hypothesis that the bimodal activity in DD observed by us is influenced by the gradual LD.

In order to understand how seminatural temperature cycles could affect the activity and clock gene expression in *Ae. aegypti*, we combined seminatural TC with constant darkness conditions. This was sufficient to entrain activity as it normally occurs under the traditional rectangular temperature cycles, but with the benefit of not producing the artifacts (masking) after abrupt changes of temperature. Regarding the activity peaks, unlike *D. melanogaster* (Yoshii et al., 2009; Bywalez et al., 2012), we did not observe a clear indication of the M peak in mosquitoes subjected to seminatural or rectangular temperature cycles with constant darkness (Figures 3A,B, 4). We also observed that the E peak occurred at a different time in seminatural TC in comparison to the rectangular TC (Figures 3A–C, 4). This peak occurs earlier in rectangular TC (ZT6) than in seminatural TC (ZT8), while it is coinciding under gradual LD conditions (ZT12) or rectangular LD conditions (ZT12) (Figures 3A–C, 4; Gentile et al., 2009).

It is also important to note that the E peak is controlled by clock synchronization effect and it is not just a masking in LD. This is because the phase of this peak persists quite similarly in the early days of DD, compared to activity when mosquitoes were in LD (Figures 2A–C, for more details of classic chronobiology protocols, see Clements, 1999). Therefore, considering that the E peak is under circadian control, we come to believe that LD and seminatural TC promote clock synchronization with greater synergy than LD and rectangular TC. This was supported by our molecular results. We carefully compared the phases of peak and trough among the genes analyzed in DD with seminatural TC (Figure 5), rectangular LD with constant temperature (Gentile et al., 2009), and DD with rectangular TC (Rivas et al., 2018). Most of their phases in seminatural TC were similar to LD. In contrast, the majority of those under rectangular TC regimens were different from those under LD conditions (Supplementary Tables 3, 4). In addition, gene expression appeared to occur earlier in rectangular TC than in seminatural TC. This would probably justify the advance of activity in rectangular TC (Figure 3 and Supplementary Tables 3, 4).

We also observed that the M peak had low amplitude in the gradual LD with seminatural TC (Figures 6A,B). Thus, morning temperatures of about 20°C tend to inhibit the M peak of activity. It should be remembered that the seminatural TC cycles we produced represent mean variations found during the equinoxes in Rio de Janeiro. However, during the winter the minimum temperature in Rio de Janeiro falls below 20°C, while during the summer it remains almost always above these levels (according to the INMET meteorological measurements). Therefore, it seems very likely that these mosquitoes present a bimodal pattern of activity in the summer and a crepuscular profile in the winter of Rio de Janeiro. Similar to what we speculated about Rio de Janeiro, Suwannachote et al. (2009) collected less *Ae. aegypti* in the field, in the early hours of winter mornings, in a region of Thailand where the minimum temperatures are approximately 21°C.

To better assess the influence of gradual LD with seminatural TC cycles, we subjected mosquitoes to these out-of-phase environmental indicators (Figures 6A,B). Under these conditions, we observed both an increase of the M peak and a decrease of the E peak, in comparison to the LD with in-phase TC. It reinforces the importance of temperature cycles on the amplitude of activity peaks. However, the activity of *Ae. aegypti* remained diurnal even in seminatural conflicting conditions (Supplementary Figure 1C), different from what was observed in rectangular conflicting conditions (Rivas et al., 2018).

In rectangular LD with out-of-phase TC we had used the same maximum and minimum temperatures as in seminatural LD with out-of-phase TC (30 and 20°C, respectively). It was expected that, as a diurnal species, *Ae. aegypti* had a preference to be more active during the photophase. However, in this rectangular conflicting condition, the mosquitoes were exposed to 20°C during the entire photophase and it may be the reason why the daily activity of *Ae. aegypti* changed from the photophase to scotophase: as an “escape” from the low temperatures of the photophase (Rivas et al., 2018).

On the other hand, when the mosquitoes were in seminatural conflicting conditions, the minimum temperature (20°C) that normally inhibits their activity did not remain constant during 12 h. It momentarily reached this value—specifically at ZT12—which was the time of occurrence of the E peak (Figures 6A,B). Interestingly, we observed that the E peak was split into two peaks (E1 and E2 peaks). E1 advanced to ZT8.5, and E2 remained at ZT12. Consequently, the onset of the E peak occurred earlier (Figures 6A,B). Wild-type fruit flies show a similar advanced profile in LD with seminatural out-of-phase TC, but arrhythmic mutant flies did not, suggesting that the advance of evening activity onset is a clock-dependent response in *Drosophila* (Currie et al., 2009).

Based on the studies on clock genes in *Ae. aegypti* conducted by our group (Gentile et al., 2009; Rivas et al., 2018), we compared the recent results to different regimens (Supplementary Tables 3, 4). The mosquitoes seem to anticipate the phase of expression of several clock genes in the rectangular temperature cycles conditions (square symbols). This reinforces that the rectangular temperature cycles may not reflect what occurs in nature. Then, we have some questions about current research with *Ae. aegypti*. We remember that the clock of this mosquito can control several genes involved in growth, development, oviposition, immunity, response to insecticides, among other phenotypes (Ptitsyn et al., 2011; Leming et al., 2014). Thus, perhaps the use of the seminatural conditions we proposed could guarantee results in lab conditions that are closer to what occurs in the environment for several search fields.

Additionally, very little is known about the molecular pathways that lead to synchronization by temperature cycles in insects. In this study, even with the methodological limitations of using mosquitoes as a model of study, we suggest that one core clock gene plays a role in the synchronization by the temperature cycles: *timeless*. In LD with constant temperature, *tim* presented an expression peak at ZT13 (with a borderline statistical difference) (Supplementary Table 3; Gentile et al., 2009). Conversely, in DD with rectangular TC, the expression peak of this gene occurred at ZT9 (Supplementary Table 3; Rivas et al., 2018). In LD with TC out of phase, the temperature cycles start at ZT12 (here we take the lights on as a reference for ZT0). This means that in rectangular LD with TC out of phase, while the LD cycles “advised” the clock that the peak of *tim* must occur at ZT13, the temperature cycles synchronize to the ZT21 (12 + 9). Interestingly, the peak of *tim* in rectangular LD with TD out of phase occurred at ZT17, an intermediate time for the pressure of the two oscillators (Rivas et al., 2018).

We saw a different scenario in seminatural cycles. In DD with seminatural TC, as well as in LD with constant temperature, *tim* has a peak of expression at ZT13 (Figure 5 and Supplementary Table 3; Gentile et al., 2009). This is equivalent to saying that, in LD with seminatural TC out of phase, the two oscillators exert forces at perfectly antagonistic times. In other words, while the LD cycles would synchronize the *tim* peak at ZT13, the temperature cycles would adjust the peak at ZT1 (12 + 13). Just because there is a “tug-of-war” of similar forces, *tim* becomes arrhythmic in LD with seminatural TC out of phase (Figure 7 and Supplementary Table 3). However, when we did the knockdown of *nocte*, *tim* not only returned to being rhythmic but also showed a peak of expression in the ZT9. That is, with the reduction of *nocte*, the expression of this gene was closer to the synchronization exerted by the LD cycles (ZT13), than by the temperature cycles (ZT1). This strongly suggests that *nocte* has an important role in the synchronization by temperature cycles, especially on the expression of *tim* in *Ae. aegypti*. This would be similar to removing *Cry* in *Drosophila* during light/dark with temperature cycles out of phase, which made the flies follow the temperature regime rather than becoming arrhythmic (Harper et al., 2016).

Curiously, the silencing of *tim* via dsRNA affected the activity of *Aedes* (Gentile et al., 2009; Gentile et al., 2013). In *Drosophila*, the alternative splicing of *tim* has been implicated in behavioral adaptation to seasonal temperature changes (Boothroyd et al., 2007; Montelli et al., 2015). Thus, future studies about possible RNA isoforms of *tim* in *Aedes* may be an interesting line of research to understand the circadian clock of these mosquitoes.

In addition to what was observed for *tim*, we also highlight the importance of the *nocte* gene. Here, we show that *nocte* affects the levels of activity in these vectors (Figure 9). Interestingly, the silencing of *nocte* via dsRNA caused a reduction in the expression of this gene in the body, but not in the head, which was enough to affect behavior (Figures 8, 9). Curiously, in *Drosophila* different neurons of the circadian pacemaker can synchronize to temperature cycles, but in isolated brains this response does not occur, since the information is passed through the peripheral organs (Sehadova et al., 2009). The main structure capable of perceiving the thermal variations and transmitting it to the other peripheral oscillators are the chordotonal organs (Sehadova et al., 2009). As mentioned, a mutation in *nocte* causes structural and functional changes in the ChOs of *Drosophila*, hindering the synchronization of gene expression and activity by temperature cycles (Glaser and Stanewsky, 2005; Sehadova et al., 2009; Chen et al., 2018). Thus, since our injection of *nocte* dsRNA did not cause a reduction in the expression of this gene in the head of *Aedes*—only in the body—the peripheral tissues are probably crucial for the synchronization by temperature cycles through *nocte* in these mosquitoes, as occurs in *Drosophila*.

Chen et al. (2018) reported that, in *Drosophila*, *nocte* mutants show normal levels of DD rhythmicity after LD entrainment in constant temperature, but reduced DD rhythmicity after temperature entrainment. Thus, we agree that the analysis of the behavior of these mosquitoes in constant condition to estimate the free-running period and level of rhythmicity would be interesting for us to better understand the role of *nocte* in *Ae. aegypti*. However, RNAi is the method most often used in mosquitoes for functional analysis of the genes of interest but does not allow a prolonged knockdown for a considerable number of days (Gentile et al., 2013). Therefore, unfortunately, it would not be possible to estimate how the elimination of *nocte* affects *Aedes* in DD with RNAi. Future studies that use techniques such as CRISPR/Cas9 will probably be able to answer our questions. Even though, here, the role of *nocte* for the entrainment was not fully explored due to the limitations of the technique, the only condition used (LD with TC out of phase) was sufficient to reveal interesting effects of *nocte* gene knockdown on behavior (reduction of amplitude on activity) and gene expression (*tim* rhythmic expression is rescued in *nocte*-depleted mosquitoes). These results are solid in the sense of showing the importance of *nocte* for the temperature perception in mosquitoes and suggest that the role of this gene is conserved between these insects and fruit flies. Lastly, the scientific community has been speculating on the influence of temperature on the behavior of mosquitoes for decades (Clements, 1999). In this study, we finally present candidate genes that were influenced by temperature cycles and affect *Aedes* activity: *timeless* and *nocte*. Therefore, we believe that further studies involving the role of these genes in mosquitoes under different physiological conditions can contribute significantly to reveal important epidemiological aspects of these vectors.

## Data Availability Statement

The original contributions presented in the study are included in the article/Supplementary Material, further inquiries can be directed to the corresponding author.

## Author Contributions

RT, GR, RB, and AP: conceptualization and design of study. RT, GR, and RB: performance, formal analysis, writing, revision, and editing. RT: writing original draft. RB: supervision. RB and AP: funding acquisition. All authors contributed to the article and approved the submitted version.

## Conflict of Interest

The authors declare that the research was conducted in the absence of any commercial or financial relationships that could be construed as a potential conflict of interest.
