# Supplementary material for: The Summer Is Coming: nocte and timeless Genes Are Influenced by Temperature Cycles and May Affect Aedes aegypti Locomotor Activity
Source: Front Physiol. 2020 Dec 23;11:614722. doi: 10.3389/fphys.2020.614722 (PMC7786104; doi:10.3389/fphys.2020.614722)
Supplement: Supplementary file 1 [file Data_Sheet_1.pdf]

Supplementary Material

Supplementary Figures and Tables

Fig. S1.

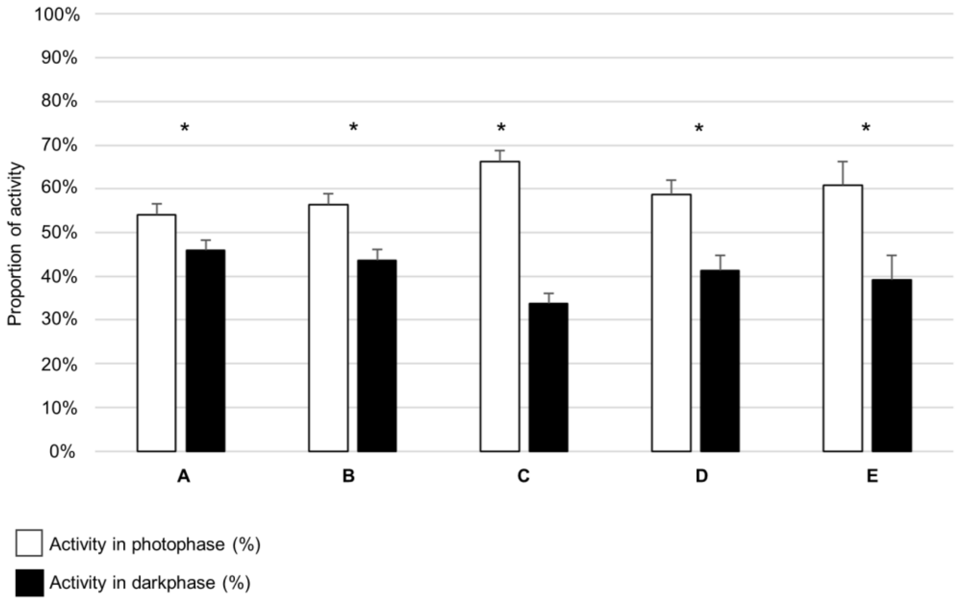

Fig. S1. **Proportion of activity during the day and the night**

(A-E) Percentage of activity in photophase (white bars, ZT0.5-12) and darkphase (black bars, ZT12.5-24) of *Aedes aegypti* in different conditions. (A) Mosquitoes in gradual LD with constant 25 °C (n = 14). (B) Mosquitoes in gradual LD with in-phase TC (n = 74). (C) Mosquitoes in gradual LD with out-of-phase TC (n = 74). (D) Mosquitoes injected with dsRNA of *LacZ* in gradual LD with out-of-phase TC (n = 18). (E) Mosquitoes injected with *nocte* dsRNA in gradual LD with out-of-phase TC (n = 13). Asterisks indicate when the difference between groups was significant according to the t-Student test, $p < 0.05$.

Fig. S2.

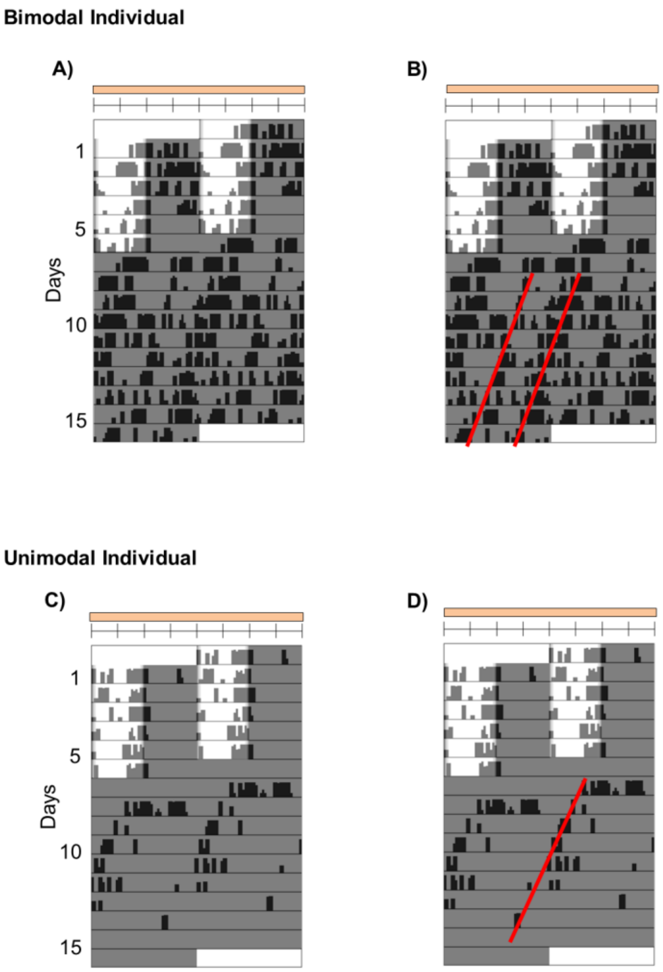

Fig. S2. Individuals with different activity profiles

(A-D) The mosquitoes were entrained by LD cycles with simulated dawn and dusk (constant temperature, 25°C) for six days. After that, they were kept in constant darkness and temperature (25°C) for 10 days. (A-B) Double-plotted actogram of the same individual with bimodal activity profile. This mosquito showed morning and evening activity peaks in constant darkness and temperature. (A) We showed the bimodal individual actogram without markers or (B) with marked peaks (red line). (C-D) Double-plotted actogram of mosquito with unimodal activity profiles. This individual exhibited only evening activity peak under constant darkness and temperature. (C) We showed the unimodal individual actogram without markers or (D) with marked peaks (red line). Area in actograms represents light/dark conditions: lights on = white, lights off = gray. Orange bars above the actograms indicate temperature regimen (25 °C).

Fig. S3.

ATGAAGATTCTCTGGCAGCATGAATGCaTTGGGGGGAACTAGGGGGGAGC
GAAATGCAAAGCCCAAATTCGCAGCGTTAGATATCAA***CAAACTCTACAGC***
ACAAGTCGG_GGAGA_ATCTCTTGAACCATCAACTCAGAAAAGTGCAGCTCC
TCGTAAACATGGAATGCAAAGTTTGGGAAAGGTTCCCTCGGCTCGTCGAC
CGCCAGCAAACCTTCCGTCTCTGAAAGCCGAAATCTCCATTCCATCGGATC
AGCAAGGCACCTGGGGTAGCGAAGCAGGAGACAACCAAAACAACAACTCT
AGTATTACTTCAACATCAGCAGCACCATCAGCAGCCGCCGGTAGCAGCAC
AAATAACAACGCCACGGTTGGTGCCGGAGGTGTGCACAGCACTTCACACG
CCAGCAGTTCACAGATTCCCCACTCGGGCGCATCATCCTCTTCACAATGG
AGTTCGAACGAGTTCCCCTcGcTGGATGGCACTGGTCAGTATGGTCCGAG
CGGCAAACAGCaGCATCATCATCTCCACGATGGCACAGACGGCAGAGCGA
TGGGTCAGTACATGGAT**GGTCCACAAGTCAGTCTGC**GGCCTCAAACGGAT
GCTGCGAGTTGGATGCAGCAACAACAGCAGAGCAGTGGGGGCGGAAACA
ACGCCGGAGGTATGAACGGGCCAAACAGCAACCAACAGGGCCAGGGCCC
CCAACAGCAACCAGCTGCACCCCTACCGCCTCAGTTCCGGGCACTGATGC
CACCGTTCATGTA

Fig. S3. Cloned coding sequence of *nocte* with 762 base pairs (bp)

ATG (grey) represents the codon of the supposed initial methionine. In red: region used to make oligonucleotides. In yellow: end of an exon. In green: beginning of an exon. Minor letters represent polymorphisms.

Gene	Sense	Name	Sequence (5’-3’)
E75	foward	5aeE75RTF01	CCTACTTTGGCATGTCCACTGAA
	reverse	3aeE75RTF01	GGTGAACTTATCGTCCTGGGTCA
cwo	foward	5aecwoRTF01	CGTCTAGGCAAGATCCGCTATCA
	reverse	3aecwoRTF01	CCAGGCAGGAGTTCATCCGATC
nocte	foward	5aenctRTF01	AATTCACCGTTAGCGTTGGCG
	reverse	3aenctRTF01	GCTGCCGACCATTGGGATC
	foward	5aenctRTF01i	TAATACGACTCACTATAGGGCAAACTCTACAGCACAAGTCGG
	reverse	3aenctRTF01i	TAATACGACTCACTATAGGGGCAGACTGACTTGTGGACC

Table S1. List of primers used in qPCR and RNAi assays in *Ae. aegypti*

		Gene								
		per	*tim*	*cry2*	*cyc*	*Pdp1*	*vri*	*Clk*	*E75*	*cwo*
DD with gradual TC	$F_{5,24}$	92.02	33.62	13.53	23.81	12.84	11.09	**1.737**	**0.77**	**0.32**
	P	<0.001	<0.001	<0.001	<0.001	<0.001	<0.001	**0.2 (ns)**	**0.59 (ns)**	**0.89 (ns)**
LD with in-phase TC	$F_{5,24}$	390.1	15.25	7.919	21.28	21.72	8.08	**2.85**	**1.12**	**0.83**
	P	<0.001	<0.001	<0.001	<0.001	<0.001	<0.001	**0.06 (ns)**	**0.39 (ns)**	**0.55 (ns)**
LD with TC in anti-phase	$F_{5,24}$	263.3	**1.75**	6.98	23.94	90.33	5.01	**2.02**	**0.43**	**0.3**
	P	<0.001	**0.18 (ns)**	<0.001	<0.001	<0.001	<0.01	**0.12 (ns)**	**0.82 (ns)**	**0.91 (ns)**

Table S2. Statistical Analysis of the Circadian Expression of Clock Genes in *Ae. aegypti*.

		ZT	-12	-8	-4	0	+4	+8	+12
per	DD with seminatural TC	17				●			
	DD with rectangular TC	13			■				
	Rectangular LD with constant temperature	17							
	Seminatural LD and TC	17				●			
	Rectangular LD and TC	13			■				
tim	DD with seminatural TC	13				●			
	DD with rectangular TC	9			■				
	Rectangular LD with constant temperature	13				▲			
	Seminatural LD and TC	13				●			
	Rectangular LD and TC	9			■				
1st peak of *cry2*	DD with seminatural TC	1				●			
	DD with rectangular TC	1				■			
	Rectangular LD with constant temperature	1				▲			
	Seminatural LD and TC	1				●			
	Rectangular LD and TC	1				■			
2nd peak of *cry2*	DD with seminatural TC	17				●			
	DD with rectangular TC	21					■		
	Rectangular LD with constant temperature	17				▲			
	Seminatural LD and TC	17				●			
	Rectangular LD and TC	17				■			
cyc	DD with seminatural TC	1				●			
	DD with rectangular TC	21			■				
	Rectangular LD with constant temperature	5					▲		
	Seminatural LD and TC	5					●		
	Rectangular LD and TC	1				■			
vri	DD with seminatural TC	9				●			
	DD with rectangular TC	9				■			
	Rectangular LD with constant temperature	9				▲			
	Seminatural LD and TC	9				●			
	Rectangular LD and TC	9				■			
Pdp	DD with seminatural TC	17				●			
	DD with rectangular TC	13			■				
	Rectangular LD with constant temperature	21					▲		
	Seminatural LD and TC	17				●			
	Rectangular LD and TC	13			■				

Table S3. Expression peaks of clock genes in different regimens

The expression profile of clock genes described in DD with rectangular TC, rectangular LD and TC (Rivas et al., 2018) and rectangular LD with constant temperature (25°C) (Gentile et al., 2009) were re-examined using the same interval of 4 hours between ZTs that we applied under DD with seminatural TC and seminatural LD and TC (i.e., ZTs 1, 5, 9, 13, 17 and 21). The third column shows the ZT where we observed the peak of gene expression in each condition. To compare the expression variation between the regimens in a homogeneous manner between the genes, the peak of expression

in all conditions was normalized with peak of expression in DD with seminatural TC (grey lines). Positive and negative signals represent delays and advances in the phases of expression peak, respectively. Delays longer than +12 are considered as phase advances. Advances less than -12 are considered as phase delays. For example, *per* has a peak expression at ZT 17 in DD with seminatural TC and at ZT 13 in DD with rectangular TC. Thus, once we used the expression in DD with seminatural TC as a normalizing profile, this gene advanced -4 ZTs in DD with rectangular TC (13 - 17 = -4). On the other hand, *cyc* has a peak of expression in ZT 1 in DD with seminatural TC and in ZT 21 in DD with rectangular TC. Thus, it is more likely this gene has advanced -4 ZTs than delayed +20 ZTs in DD with rectangular TC. Therefore, we use the correction factor -24 for indices greater than +12 and +24 for indices smaller than -12. In the last example, we would have: 21 - 1 - 24 = -4. We used the circle for conditions in which the temperature cycles were seminatural. The squares represent regimens in which the temperature cycles were rectangular and the triangle only occurs when the temperature is constant. Note that all of the genes that anticipated their peak of expression were in rectangular TC (square symbol). Since *E75* and *cwo* had not been observed by Gentile *et al*. 2009 and Rivas *et al*, 2018, we could not compare those genes. *Clk* was not considered either, because it was arrhythmic in all conditions. *tim* presented borderline statistics in LD with constant temperature and we considered it in this analysis (Gentile *et al*., 2009).

		ZT	-12	-8	-4	0	+4	+8	+12
trough of *per*	DD with seminatural TC	5				●			
	DD with rectangular TC	5				■			
	Rectangular LD with constant temperature	5				▲			
	Seminatural LD and TC	5				●			
	Rectangular LD and TC	5				■			
trough of *tim*	DD with seminatural TC	5				●			
	DD with rectangular TC	1			■				
	Rectangular LD with constant temperature	9					▲		
	Seminatural LD and TC	5				●			
	Rectangular LD and TC	1			■				
trough of *cry2*	DD with seminatural TC	9				●			
	DD with rectangular TC	5			■				
	Rectangular LD with constant temperature	9				▲			
	Seminatural LD and TC	9				●			
	Rectangular LD and TC	9				■			
trough of *cyc*	DD with seminatural TC	13				●			
	DD with rectangular TC	9			■				
	Rectangular LD with constant temperature	13				▲			
	Seminatural LD and TC	13				●			
	Rectangular LD and TC	13				■			
trough of *vri*	DD with seminatural TC	1				●			
	DD with rectangular TC	21			■				
	Rectangular LD with constant temperature	21			▲				
	Seminatural LD and TC	21			●				
	Rectangular LD and TC	1				■			
trough of *Pdp*	DD with seminatural TC	5				●			
	DD with rectangular TC	1			■				
	Rectangular LD with constant temperature	5				▲			
	Seminatural LD and TC	5				●			
	Rectangular LD and TC	5				■			

Table S4. Expression trough of clock genes in different conditions

The whole process of analysis and reference of each condition was conducted exactly as described in table S.2, with the only difference that here we described the expression trough of the genes. The third column exhibits the corresponding ZT to the trough of gene expression in each condition. Note that the majority of the genes that anticipated their expression trough were in rectangular TC (square symbol). Since *E75* and *cwo* had not been observed by Gentile *et al*, 2009 and Rivas *et al*, 2018, we could not compare them. *Clk* was not considered either, because it was arrhythmic in all conditions. *tim* presented borderline statistics in LD with constant temperature and we considered it in this analysis (Gentile *et al.*, 2009).

Supplementary references

1. Gentile, C., Rivas, G.B.S, Meireles-Filho, A.C., Lima, J.B.P. and Peixoto. A.A. (2009). Circadian expression of clock genes in two mosquito disease vectors: *cry2* is different. J Biol Rhythms 24, 444-451. doi: 10.1177/0748730409349169.

2. Rivas, G.B.S., Teles-de-Freitas, R., Pavan, M.G., Lima, J.B.P., Peixoto, A.A. and Bruno, R.V. (2018). Effects of Light and Temperature on Daily Activity and Clock Gene Expression in Two Mosquito Disease Vectors. J Biol Rhythms. 33, 272-288. doi: 10.1177/0748730418772175.
